# Analysis of the Productivity, Immunity, and Health Performance of Nile Tilapia (*Oreochromis niloticus*) Broodstock-fed Dietary Fermented Extracts Sourced from *Saccharomyces cerevisiae* (Hilyses): A Field Trial

**DOI:** 10.3390/ani11030815

**Published:** 2021-03-14

**Authors:** Nermeen M. Abu-Elala, Tamer El-Sayed Ali, Naela M. Ragaa, Sara E. Ali, Reham M. Abd-Elsalam, Nehal A. Younis, Dalia A. Abdel-Moneam, Aya H. Hamdien, Melina Bonato, Mahmoud A.O. Dawood

**Affiliations:** 1 Department of Aquatic Animal Medicine and Management, Faculty of Veterinary Medicine, Cairo University, Giza 12211, Egypt; nabouelkaramat@gmail.com (N.A.Y.); dr.daliaashraf@googlemail.com (D.A.A.-M.); 2 Department of Oceanography, Faculty of Science, Alexandria University, Alexandria 21568, Egypt; tameraly@yahoo.com (T.E.-S.A.); aya24hesham@gmail.com (A.H.H.); 3 Department of Nutrition and Clinical Nutrition, Faculty of Veterinary Medicine, Cairo University, Giza 12211, Egypt; nalamohamed@gmail.com; 4 Department of Physiology, Faculty of Veterinary Medicine, Cairo University, Giza 12211, Egypt; sara.physiology@gmail.com; 5 Department of Pathology, Faculty of Veterinary Medicine, Cairo University, Giza 12211, Egypt; reham_pathology@hotmail.com; 6 Research and Development, ICC Industrial Comércio Exportaçãoe Importação SA, São Paulo 01451-909, Brazil; melina.bonato@iccbrazil.com.br; 7 Department of Animal Production, Faculty of Agriculture, Kafrelsheikh University, Kafrelsheikh 33516, Egypt

**Keywords:** functional feed, seeds, production, mortality, growth, immune response

## Abstract

**Simple Summary:**

The low performance of Nile tilapia (*Oreochromis niloticus*) broodstock and high seed mortality along the spawning season are the major constraints which are directly reflected in hatchery profit. Broodstock nutrition is a key factor that can influence fish reproduction and subsequent larval quality. The objective of this study was to evaluate the effect of dietary fermented extracts sourced from *Saccharomyces cerevisiae* (nucleotides, β-glucans and MOS) (Hilyses^®^) on the seed production and health of Nile tilapia broodstock, as well as on seed survival and growth performance. The study was performed in the hatchery along the spawning season and continued in the laboratory to monitor the performance in fry and fingerlings. The results showed that dietary fermented yeast extracts could be used as a strategic approach to sustain tilapia production, as they improve the productivity and health of broodstock as well as seed survival and performance.

**Abstract:**

The present study aimed to evaluate the effect of dietary fermented extracts sourced from *Saccharomyces cerevisiae* (nucleotides, β-glucans and MOS) (Hilyses^®^) on the production and health of Nile tilapia (*Oreochromis niloticus*) broodstock, as well as on seed survival and performance. The trial was performed in a hatchery along the spawning season and continued in the laboratory to monitor the performance in fry and fingerlings. The broodstock were divided into two groups, (C) fed a basal diet and (H) fed 0.4% Hilyses. Blood and histological parameters, antioxidant power, cortisol level and the expression of some immune-related (*TLR-2*, *IL-1β* and *TNF-α*) and growth-related genes (*MUC-2* and *IGF-1*) were measured. The obtained seeds were subdivided into four treatments: (C-C) fed a basal diet, (C-H) fed 0.4% Hilyses, (H-C) fed a basal diet and (H-H) fed 0.4% Hilyses. Results revealed that the dietary inclusion of Hilyses in the broodstock increased seed production, survival, hematological parameters, and antioxidant power. Moreover, it improved the intestinal microstructure and upregulated the immune- and growth-related genes. The growth indices of fry and fingerlings were significantly increased in all Hilyses-treated groups (*p* < 0.05). The performance in the (H-H) group significantly surpassed those of all groups. Therefore, dietary fermented yeast could be used as a strategic solution to sustain tilapia production.

## 1. Introduction

Fish farming in Egypt represents the largest part of production compared to captured fish and can reach up to 77% of the total production; the private sector share of this production is more than 99% [1]. Although several factors limit aquaculture development (e.g., feeding cost, diseases, bad water quality, low performance of broodstock, and the high mortality rate in seeds) [2], Egypt has the largest aquaculture industry in Africa and the third largest after China and Indonesia in Nile tilapia production [3,4]. Global tilapia culture has witnessed a sharp expansion during the past two decades and is being cultured in more than 130 countries worldwide. Tilapias are currently the second most important farmed finfish in the world, representing 125% of freshwater fish production and 107% of total fish culture [4]. Global production of farmed tilapia grew by 3.3 percent in 2020 to top 6 million tons for the first time, despite the impact of COVID-19 [5]. The expansion of tilapia production all over the world is due to its ability to be produced in various aquatic environments, selective breeding, and its potential to replace marine fish products [5]. There are several strategic approaches that have been recently adopted in aquaculture to sustain tilapia production. One of these approaches is the production of functional foods. A wide range of functional feed additives have been used [6]; certain additives, such as pellet binders, antimicrobials, antimycotoxins, antioxidants, and enzymes, act by improving feed quality. Other additives, such as probiotics, prebiotics, phytogenics, dietary acidifiers and immunostimulants, directly affect animal performance and health [6,7,8]. Functional feeds containing gut health promotors have become a key component of any strategy to prevent diseases in aquaculture, particularly when opportunistic bacteria are suspected to be a major cause of mortality [9,10]. Gut health promotors combine different modes of action, including modulating the intestinal microbial flora, stimulating the local immunity of the gut and improving the histological intestinal structure [11]. This approach directly correlates with the feed transformation into biomass gain and the profit of the farm.

*Saccharomyces cerevisiae* and its byproducts are the most common functional feed additives used in aquaculture [6]. Hydrolyzed, fully fermented yeast contains numerous nutrients and bioactive compounds, e.g., peptides, polypeptides glutamines, β-glucans, mannan oligosaccharides (MOS), chitin and nucleotides [12]. These compounds have different mechanisms of action and show benefits when used alone or in combination. Briefly, β-glucans are known by their immunostimulatory effect; they stimulate the innate immunity and enhance the resistance of fish and crustaceans against microbial diseases [13]. MOS is characterized by pathogen adsorption capacity, preventing the bacterial colonization, and stimulating the proliferation of beneficial gut flora. In addition, they increase the food palatability and improve the intestinal health [14]. Nucleotides are the building block of cell’s RNA. They are synthesized de novo in most tissues, but some tissues like, intestinal cells, hepatocytes, hemolymph, and immune cells have limited capacity for synthesis and need exogenous supply, especially under certain conditions, e.g., tissue injury, liver dysfunction, diseases, reproduction, and fast growth life stage [12]. Essential amino acids, such as glutamic acid, play a substantial role in tissue proliferation and regeneration [10].

Broodstock nutrition is recognized as a major factor that can influence fish reproduction and subsequent larval quality [15]. Many studies have indicated the positive effects of dietary fortification of whole yeast and/or its by-products on the growth performance, hematological indices, antioxidant defense systems, immune response, and disease resistance in Nile tilapia [9,16,17,18,19]. Abu-Elala et al. [5] recorded that dietary fermented yeast extracts sourced from *Saccharomyces cerevisiae* successfully enhanced the phagocytic activity/index, and lysozyme activity up-regulated the proinflammatory cytokines (*TNF-α* and *IL-1β*) and increased the resistance of fish against bacterial diseases. In addition, they improved the oxidant and antioxidant defense systems and reduced the lipid peroxidation. Moreover, authors observed a significant increase in RBCs count, WBCs count, Hb, and hematocrit, which could be related to the hepato-stimulatory and hepatoprotective effects of yeast by-products. Despite the progress made with tilapia growth and immunity in fry, fingerlings, and grow-out stages, few studies have examined the effects of additives on the performance, immunity, and health of broodstock and the subsequent effects on tilapia fry production and survival [16,17,18]. So, the current study is needed to fill the gaps regarding the potential effects of fermented yeast products (Hilyses, ICC Brazil) rich in nucleotides, β-glucans, MOS and essential amino acids to support the performance and productivity of Nile tilapia broodstock during the spawning season (from March until August) under prevailing Egyptian conditions. The hematological indices, antioxidant capacity and cortisol levels in broodstock were measured at the end of the spawning season. In Addition, the histological findings and relative expression of immune and growth-related genes e.g., toll like receptor *TLR-2*, tumor necrosis factor α *TNF-α*, interleukin *IL-1β*, insulin growth factor *IGF-1*, and mucin *MUC-2* were assessed. Furthermore, we evaluated the influence of Hilyses-treated broodstock and/or Hilyses-treated diets on offspring survival and growth performance.

## 2. Materials and Methods

### 2.1. Hilyses Composition and Diet Preparation

Hilyses (Hilyses, ICC Industrial Comércio Exportaçãoe Importação SA, São Paulo, Brazil) is a commercial hydrolyzed fermented yeast product with 63 g/kg nucleotides, 235 g/kg β-glucans and 142 g/kg MOS. Three experimental diets (Table 1) were used along the feeding trial: a broodstock diet (Diet 1) with 36% crude protein and 460.67 Kcal GE/100 g diet. The feed ingredients were ground in a hammer mill (SWFL170, FAMSUN, Yangzhou Jiangsu, China), filtered through a 0.5 mm sieve, and pelletized to 3 mm in diameter. Broodstock were fed the experimental diets up to the satiation level twice daily (08:00 and 15:00).

Fry diet (Diet 2) (40% crude protein and 466.08 Kcal GE/100 g diet) was used during the nursery period and the first month of the laboratory experiment, and it was in powder form. Larvae were fed Diet 2 for 30 days seven times daily up to satiation level. A fingerling diet (Diet 3) of 30% crude protein and 454.46 Kcal GE/100 g diet, in a pelleted form with 1 mm diameter, was used in the second growth interval (30 days). From day 31 till day 90, fry and fingerlings fed the test diets up to the satiation level 3 to 5 times daily. All three experimental diets were formulated to satisfy the nutrient requirements of *O. niloticus* broodstock, larvae, fry and fingerlings [19], and Hilyses was added to the experimental diets (Diets 1, 2, and 3) at 0.4% during the coating stage of the pellets.

### 2.2. Experimental Design

This study was performed through two experiments:

#### 2.2.1. Hatchery Trial: Evaluating the Effect of Dietary Inclusion of Hilyses on Production, Seed Mortality and Immune Response of Nile tilapia Broodstock

The hatchery phase began in mid-February. A total of 880 females (250 ± 26.3 g) and 352 males (400 ± 42.6 g) of Nile tilapia broodstock were netted from earthen ponds, selected manually, sexed, and transferred to conditioned concrete tanks under a greenhouse, where they were held and kept separately for 20 days for adaptation to the new environment.

The broodstock were divided into two treatment groups with 11 tanks for each, 40 females and 16 males/tank. Group (H) was fed a Hilyses-supplemented diet at 0.4%, and group (C) was fed a control diet (Table 1). Natural mating was practiced by placing the broodstock in breeding tanks (3 × 8 × 1 m^3^) and mating at a sex ratio of 1:3 (male: female). On the 12th day, broodstock were collected and transferred to earthen ponds for 3 weeks of rest and feeding. Then, tilapia seeds were collected, counted, and transferred to nursery ponds.

This experiment lasted for 6 months from March until August. In March and April, the water temperature was adjusted to 28 °C with a heater, and the water exchange rates varied according to the temperature outside the greenhouse. From May until August, the environmental temperature rose and reached more than 30 °C in a greenhouse. The environmental water parameters were measured daily and are recorded in Table 2.

##### Blood and Tissue Samples

At the end of ‘hatchery experiment, ten fish from each broodstock group were anaesthetized with commercial clove oil at 1 mL/10 L water (Ectyo-colve^®^, Deneuille-Les-Mines, France). Whole blood and serum samples were collected from the caudal vessels for hematological, biochemical, total antioxidant capacity and cortisol measurements. Samples from the intestine (middle part) and liver tissues were collected and stored at −80 °C until analysis for measuring the malondialdehyde (MDA) and glutathione reduced (GSH) levels and for relative expression of immune-related and growth-related genes. Samples from the liver, intestine, kidney, and spleen were collected at the end of the hatchery experiment and preserved in 10% formalin for histological evaluation.

##### Hematological and Biochemical Analysis

The hematocrit (HTC) % was estimated according to Dacie and Lewis [20]. The hemoglobin (Hb) concentration was determined using commercial kits (Biodiagnostic Company, Giza, Egypt) according to the method of Stoskopf [21]. Erythrocytes (RBCs) were counted manually using a hemocytometer counting chamber according to the method of Stoskopf [21]. Blood indices; MCV, MCH and MCHC were calculated following Dacie and Lewis [22]. Differential leukocytic counts were estimated using the method of Shaw [23]. One hundred cells were counted to determine the different types of leukocytes (granulocytes, lymphocytes, and monocytes) using an oil-immersion light microscope at 1000× magnification.

Biochemical parameters were determined using commercial kits supplied by Spectrum (Obour City, Egypt). The activities of the liver enzymes alanine aminotransferase (ALT) and aspartate aminotransferase (AST) were evaluated according to Lopes-Virella, et al. [24], the total protein level was determined using the Biuret method according to Tietz [25], the albumin level was estimated following the method of Tietz [26], and the globulin level was calculated by subtracting the albumin results from the total protein values according to Coles [27]. For kidney function tests, the urea and creatinine levels were measured following the methods of Tietz [25] and Tietz [26].

##### Antioxidant Biomarkers, Lipid Peroxidation and Cortisol Levels Measurements

Total antioxidant capacity (TAC) was determined calorimetrically in serum using a commercial kit (Biodiagnostic Company, Giza, Egypt) according to Koracevic, et al. [28]. Reduced glutathione (GSH) activity and malondialdehyde (MDA) levels were estimated in both the liver and intestine. The samples were homogenized in 5 mL of cold buffer (50 mM potassium phosphate, pH 7.5. 1 mM EDTA) for GSH and 50 mM potassium phosphate, pH 7.5, for MDA per gram tissue using an electric tissue homogenizer (DAIHAN Scientific Co., Ltd., Wonju-si, Korea). Then, the homogenates were centrifuged for 15 min at 1800× *g*. The supernatants were collected and used to measure the GSH activity and MDA levels using commercial kits (Biodiagnostic Company, Giza, Egypt) according to Beutler et al. [29] and Ohkawa, et al. [30], respectively. The serum cortisol levels were measured following the instructions of the VIDAS^®^ Cortisol S Kit (Biomerieux. Biotechnology Company, Marcy-l’Étoile, France).

##### The Relative Expression of Immune- and Growth-Related Genes

RNA extraction from the tissue samples (liver and intestine) was applied using a QIAamp RNeasy Mini kit (Qiagen, GmbH, Düsseldorf, Germany) when 100 mg of the tissue sample was added to 600 µL of RLT buffer containing 10 μL of β-mercaptoethanol per 1 mL. For homogenization of the samples, tubes were placed into the adaptor sets, which are fixed into the clamps of the Qiagen TissueLyser. Disruption was performed in a 2-min high-speed (30 Hz) shaking step. One volume of 70% ethanol was added to the cleared lysate, and the steps were completed according to the Purification of Total RNA from Animal Tissues protocol of the QIAamp RNeasy Mini kit (Qiagen, GmbH, Düsseldorf, Germany). On-column DNase, digestion was performed to remove the residual DNA. Primers for the elongation factor *EF-1α* (housekeeping gene), insulin growth factor *IGF-1*, toll-like receptor *TLR-2*, mucin *MUC-2*, tumor necrosis factor *TNF alpha* and interleukin *IL-1β* genes used were supplied by Metabion (Germany). The primer sequences are listed in Table 3 [31,32,33,34]. Primers were utilized in a 25-µL reaction containing 12.5 µL of 2× QuantiTect SYBR Green PCR Master Mix (Qiagen, Germany, GmbH), 0.25 µL of RevertAid Reverse Transcriptase (200 U/µL) (Thermo Fisher Scientific, Inchinnan, UK), 0.5 µL of each primer at a 20 pmol concentration, 8.25 µL of water, and 3 µL of RNA template. The reaction was performed in a Stratagene MX3005P real-time PCR machine (Agilent Technologies, Santa Clara, CA, USA). The Stratagene MX3005P software version 4.10 determined the amplification curves and Ct values. For determination of the variation in gene expression with the RNA from the different samples, the CT of each sample was compared with that of the positive control group according to the “^ΔΔCt^” method described by Yuan et al. [35].

##### Histological Examination

For histological examination, samples from the liver, kidneys, spleen, and intestine were collected from both broodstock groups. The samples were fixed in 10% neutral buffered formalin (100 mL distilled water, 4 g sodium dihydrogen orthophosphate (monohydrate), 6.5 g disodium hydrogen orthophosphate (anhydrous), 100 mL formalin and 800 mL water), dehydrated using different series of ethyl alcohol (70% to 95% to 100%) and cleaned using xylene. The specimens were embedded in blocks of paraffin wax then sectioned using a microtome (Leica RM2125RTS, Leica Biosystems, Buffalo Grove, IL, USA) to obtain 3–4 µm tissue sections. The tissue sections were deparaffinized by running them in xylene to alcohol to water. Finally, the tissue sections were stained with stain hematoxylin and eosin (H&E) then examined under a microscope Leica Qwin 500 Image Analyzer (Leica, Cambridge, UK) [36]. The intestines of both groups were stained with Alcian blue-periodic acid-Schiff (PAS) staining to evaluate the acid and neutral mucin of goblet cells. In the morphometric analysis, the villi length and the crypt depth were analyzed. The villi length was calculated from tip to base, and the crypt depth was calculated from the base of the villi to the base of the crypt. In each group, ten fish were examined, and in each fish, 3 sections were examined. The heights of 5 villi and the depths of 5 crypts were measured per section.

#### 2.2.2. The Laboratory Experiment: Evaluating the Effect of Hilyses Treated (Diet and Broodstock) on the Growth Performance and Feed Utilization of Nile Tilapia Fry

Seeds obtained from the two broodstock groups (H and C) were subjected to 2 × 2 factorial arrangements: two broodstocks (broodstock fed a control diet and broodstock fed the Hilyses diet) and two diets (0.4% Hilyses and basal diets). There were five replicates per group in nursery tanks (number of seeds = 50,000 seeds/pond in a 3 × 8 m concrete tank). Seeds obtained from the broodstock fed a basal diet (C) were divided into two treatments: treatment 1 (C-C) fed the basal diet, and treatment 2 (C-H) fed the basal diet supplemented with 0.4% Hilyses. Additionally, seeds obtained from the Hilyses-treated broodstock (H) were divided into two treatments: treatment 3 (H-C) fed a basal diet, and treatment 4 (H-H) fed a basal diet supplemented with 0.4% Hilyses. After 21 days of the nursery period, fry were collected and counted again to calculate the mortality percent over 6 months.

**Figure 1:**
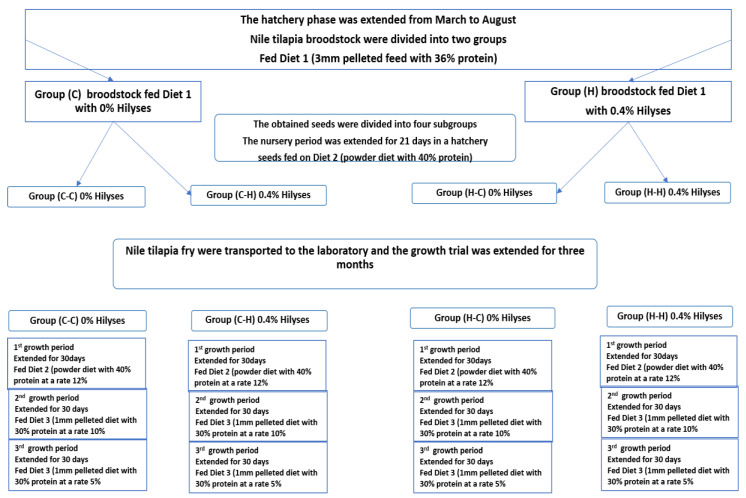
Summary of experimental design.

The laboratory phase was performed to evaluate the growth performance and feed utilization of Nile tilapia fry. The previously mentioned four treatment groups (C-C, C-H, H-C, H-H) with an average body weight of 118.8 ± 1.3 mg were allocated to 12 aquaria (30 × 40 × 100 cm) in triplicate at 20 fry/replicate. The aquaria were supplemented with aeriation and a thermostable thermometer adjusted to 26 °C. They were fed three times/day for three months [37]. Figure 1 is a summary of experimental design.

### 2.3. Statistical Analyses

Statistical analysis for broodstock hematological and biochemical parameters was performed using independent-samples *t* test, while seed production, seed mortality and growth performance data were subjected to two-way analysis of variance (ANOVA) with type of diets, time and/or broodstock as factors. Where an effect was significant, ANOVA was followed by Tukey’s HSD test for mean multi-comparison test. Significance was determined at *p* < 0.05. All statistical analysis was performed using SPSS (Version 21).

## 3. Results

### 3.1. Production of Nile Tilapia Broodstock and Percentage Mortality in Fry at the Hatchery during the Spawning Season

**Figure 2:**
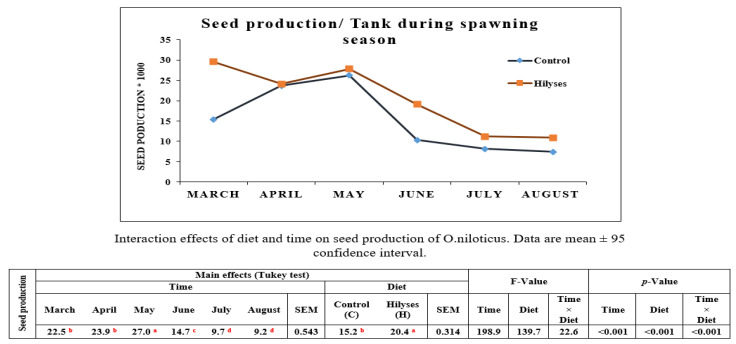
Seed production in tilapia broodstock fed a Hilyses (H) and basal diet (C) during the spawning season from March to August. The cement tank area was 3 × 8 m^2^ stocked with 40 females/16 males in each tank (*n* = 11 tanks), Temp. = 30 ± 1 °C, pH = 8.6 ± 0.2. The results were statistically analyzed using two-way analysis of variance (ANOVA) with type of diets and time as factors, followed by Tukey’s HSD test for mean multi-comparison test. Significance was determined at *p* < 0.05. ^a, b, c, d^ Means within a row with different superscripts are significantly different (*p* < 0.05).

Figure 2 depicts the effect of both Hilyses supplementation and time on the seed production during the spawning season (March to August). Overall, the data revealed that the main effects of both factors Hilyses supplementation and time were significant (*p* < 0.05) for seed production of *O. niloticus* broodstock. Moreover, the interaction between the two factors was significant (*p* < 0.05). Generally, seed production is gradually increased at the beginning of the spawning season (March to May) followed by a sharp decrease in June, then maintaining the plateau till the end of the spawning season. Whereas Hilyses supplementation to broodstock diet during the spawning period caused a gradual decrease of seed production over the period from May to July. By the end of that period, a higher level than that of the control group was shown in August.

**Figure 3:**
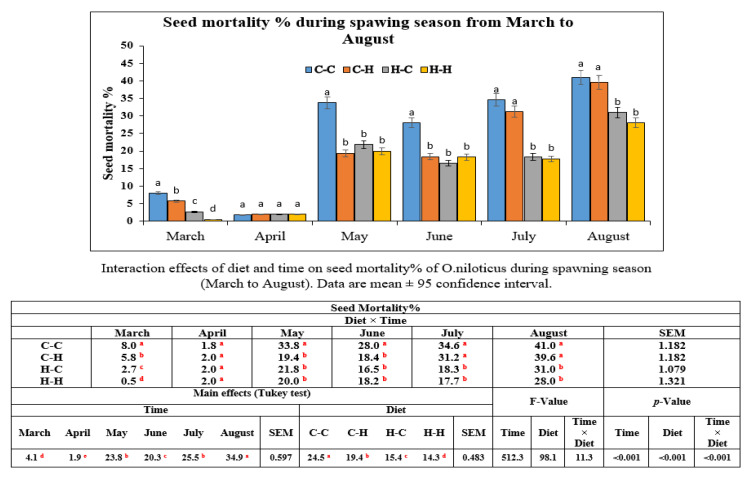
Mortality in Hilyses and control groups over the period of six months (spawning season) starting from March to August. The results were statistically analyzed by using two-way analysis of variance (ANOVA) with type of diets and time as factors, followed by Tukey’s HSD test for mean multi-comparison test. Significance was determined at *p* < 0.05. ^a, b, c, d^ Means with different superscripts are significantly different (*p* < 0.05).

Regarding the seed mortality of *O. niloticus* during the spawning season, the results are illustrated in Figure 3. At first glance, the data showed that the main effects of both factors Hilyses supplementation and time were significant (*p* < 0.05) for the seed mortality of *O. niloticus* broodstock. Additionally, the interaction between the two factors was significant (*p* < 0.05). There is a direct relationship between time and seed mortality: the percentage of seed mortality increases over the period from May to August. Hilyses had the ability to decrease the percentage of seed mortality in all supplemented groups (C-H, H-C, and H-H) compared with C-C group over the period from May to June. Moreover, at the period from July till the end of spawning season the effect of Hilyses on seed mortality was clear in (H-C and H-H) groups rather than (C-H) group which had mortality % nearly the same as that of (C-C) group.

### 3.2. Hematological and Biochemical Parameters

The Hilyses-treated group showed a significant increase in erythrocyte count, hematocrit value, hemoglobin concentration and blood indices, such as the mean corpuscular volume (MCV) and mean corpuscular hemoglobin (MCH), compared to the control group. However, there was no significant difference in the mean corpuscular hemoglobin concentration (MCHC) and white blood cell count between the groups. The differential leukocyte count displayed a significant rise in lymphocyte % at the expense of granulocyte % in Hilyses group in comparison to control ones (Table 4). Regarding the results of the biochemical analyses, both groups revealed normal liver and kidney function (Table 5).

### 3.3. Oxidant and Antioxidant Biomarkers and Cortisol Level

**Figure 4:**
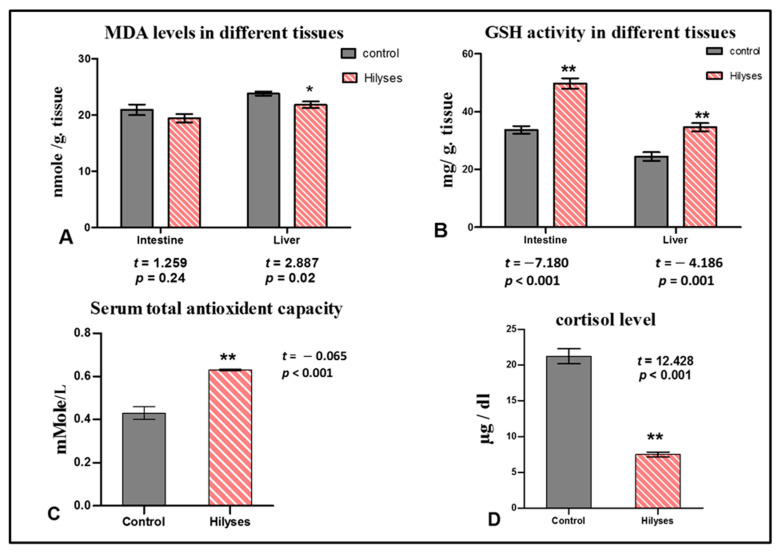
Oxidant and antioxidant biomarkers and cortisol levels in both broodstock groups. (**A**). Malondialdehyde lipid peroxide (MDA) levels in the intestine and liver of the control and Hilyses groups; (**B**). Intestinal and hepatic reduced glutathione (GSH) activity of the control and Hilyses groups; (**C**). Serum total antioxidant capacity of the control and Hilyses groups; (**D**). Cortisol activity of the control and Hilyses groups. The results were statistically analyzed by independent samples’ *t* test and expressed as the mean ± S.E. (*n* = 10). * statistically significant (*p* < 0.05), ** Statistically highly significant (*p* < 0.01) compared to the control group.

There was a significant increase in the serum total antioxidant capacity and GSH activity in the liver and intestinal tissues of the Hilyses-treated broodstock compared to the controls (Figure 4B,C). The MDA activity showed no significant difference between the groups in intestine (*p* > 0.05) while, in liver, they significantly reduced in Hilyses group when compared to control group (*p* < 0.05) (Figure 4A). Furthermore, the Hilyses-treated broodstock showed a significant reduction in cortisol levels versus the controls (Figure 4D).

### 3.4. Effect of Hilyses on Immune-Related and Growth-Related Gene Expression

**Figure 5:**
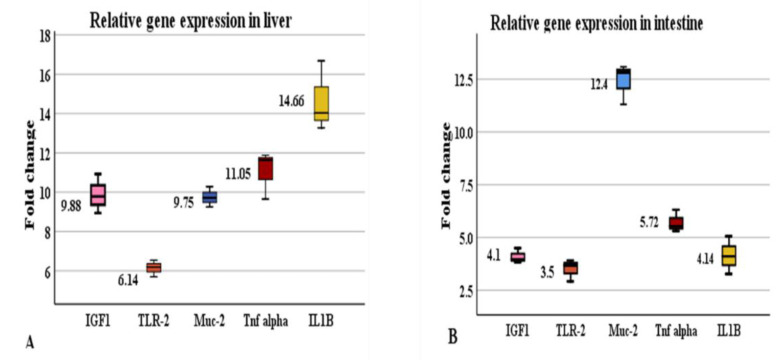
Relative expression of *IGF1*, *TLR-2*, *Muc-2*, *TNF alpha* and *IL-1β*. (**A**) gene expression of liver samples of Hilyses-treated fish (H): *IGF1* (*p* = 0.019), *TLR-2* (*p* = 0.010), *Muc-2* (*p* = 0.008), *TNF alpha* (*p* = 0.016), and *IL-1β* (*p* = 0.034) are up-regulated (*p* < 0.05) in comparison to control group (C); (**B**) gene expression of intestinal samples of Hilyses-treated fish (H): *IGF1* (*p* = 0.015), *TLR-2* (*p* = 0.011), *MUC-2* (*p* = 0.040), *TNF alpha* (*p* = 0.019), and *IL-1β* (*p* = 0.010) are up-regulated (*p* < 0.05) in comparison to control group (C). Data are represented as means ± S.E. (*n* = 10).

The expression of genes is represented as the fold increase in Hilyses treated group in comparison to control group. Genes such as *IGF-1*, *TLR-2*, *MUC-2*, *TNF-α* and *IL-1β* were upregulated in the liver and intestine tissues in response to Hilyses supplementation (Figure 5A,B). The highest expression levels of *IGF-1*, *TLR-2*, *TNF-α* and *IL-1β* were detected in liver (*p* < 0.05), while the highest expression level of *MUC-2* was observed in the intestine.

### 3.5. Histological Findings

**Figure 6:**
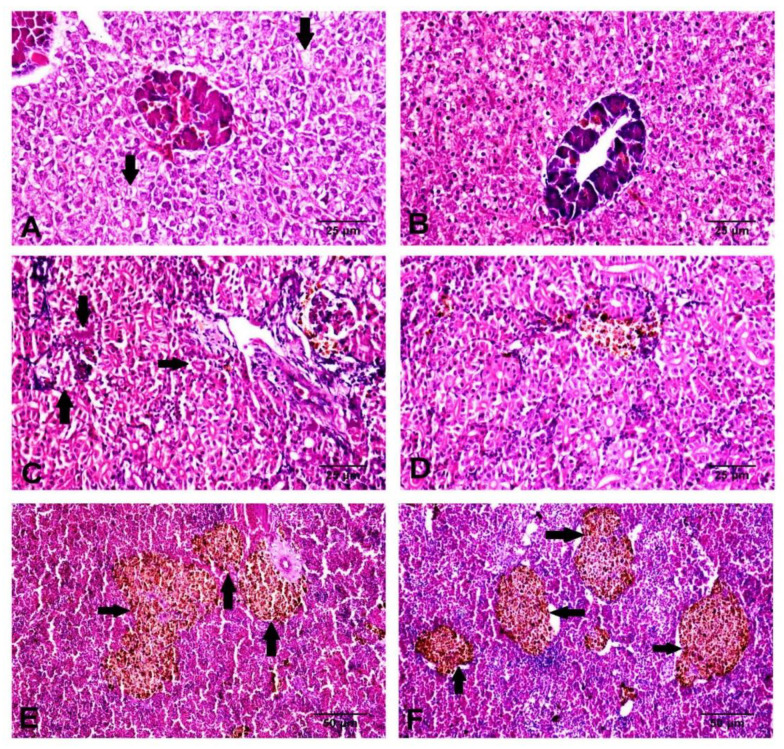
Histological examination of the liver, kidney, and spleen in the control and Hilyses-treated groups. (**A**). Liver of the control group showing vacuolation of hepatocytes (arrow) (H&E ×400); (**B**). Liver of the Hilyses-treated group showing normal histological characteristics of hepatocytes (H&E ×400); (**C**). Kidney of the control group showing necrosis of some renal tubular epithelium (arrow) (H&E ×400); (**D**). The Hilyses-treated group showing normal histological architecture of the kidney (H&E ×400); (**E**). Spleen of the control group showing moderate activation of MMCs (arrow) (H&E ×200); (**F**). Spleen of the treated group showing marked activation of MMCs (arrow) (H&E ×200).

**Figure 7:**
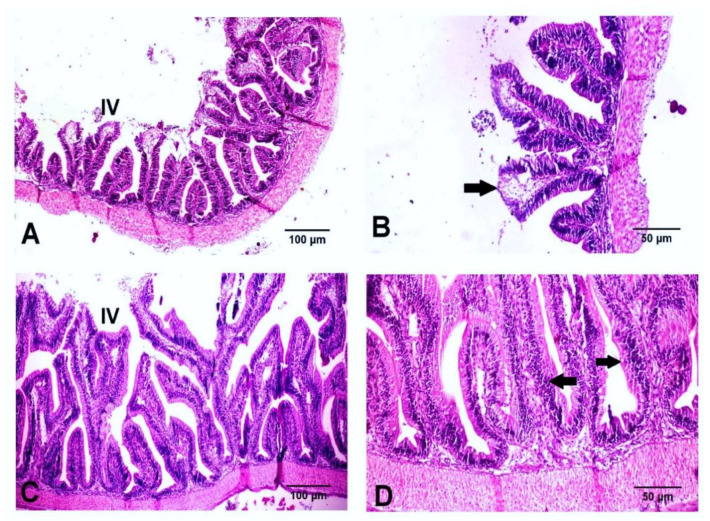
Histological examination of the intestines of the control and Hilyses-treated groups. (**A**). Control group showing intestinal villi (IV) (H&E ×100); (**B**). Higher magnification of the previous picture showing sloughing and necrosis of some enterocytes (arrow) (H&E ×200); (**C**). Hilyses-treated group showing intact intestinal villi (IV) (H&E ×100); (**D**). Hilyses-treated group showing an intestine with severe hyperplasia of enterocytes (arrow) (H&E ×200).

**Figure 8:**
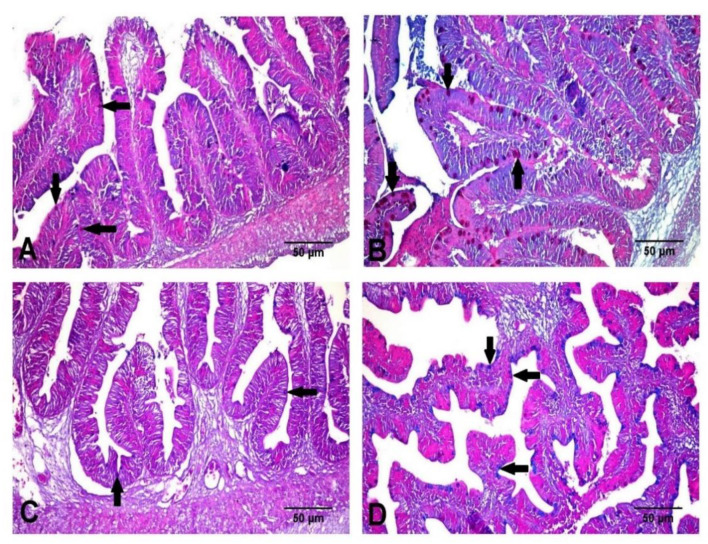
Histological examination of the intestines of the control and Hilyses-treated groups stained with Alcian blue-PAS techniques (×200). (**A**). Control group showing few goblet cells containing neutral mucins (arrow); (**B**). Hilyses-treated group showing numerous goblet cells containing neutral mucins (arrow); (**C**). Control group showing small numbers of goblet cells containing acid mucins (arrow); (**D**). Hilyses-treated group showing large numbers of goblet cells containing acid mucins (arrow).

**Figure 9:**
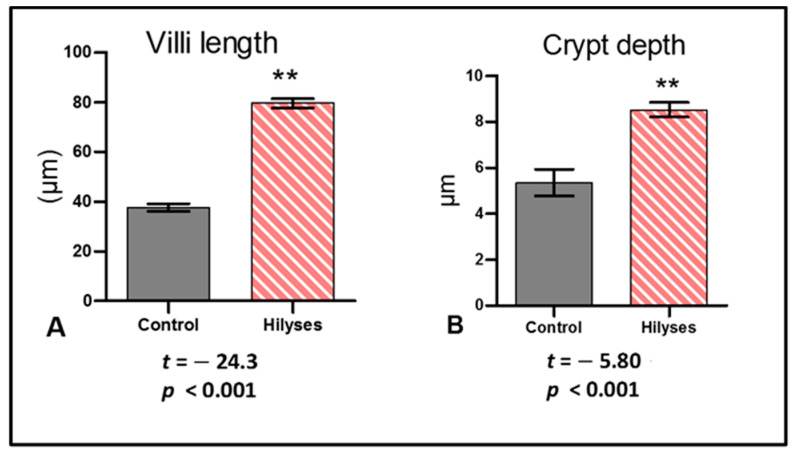
Morphometric analysis of the intestine. (**A**). Villi length; (**B**). Crypt depth. The results are expressed as the mean ± S.E. (*n* = 10) and were analyzed statistically by independent samples *t* tests, ** statistically highly significant (*p* < 0.01).

The control group showed a normal histological architecture of the liver, with mild to moderate vacuolar degeneration of hepatocytes (Figure 6A), while the treated group showed mild vacuolation of hepatocytes (Figure 6B). The kidneys revealed mild vacuolar degeneration of renal tubular epithelium with individual cell necrosis, mild depletion of hemopoietic elements and mild activation of melano-macrophage centers MMCs in the control group (Figure 6C). However, the treated group showed a normal histological structure with moderate activation of MMCs (Figure 6D). The spleen showed moderate activation of MMCs (Figure 6E), slight depletion of hemopoietic elements and lymphoidal necrosis. The treated group showed normal hepatic, renal and splenic architecture with marked activation of MMCs (Figure 6F). The intestines of both groups revealed intact mucosal lining of the intestinal villi, with some necrotic enterocytes observed in the control groups (Figure 7). In Alcian blue–PAS-stained intestinal sections, acid mucins-stained bright blue, while neutral mucins stained pink or purple in color. An intermediate color represents mixtures of the two types of mucin. The number of goblet cells stained bright blue with Alcian blue (Figure 8C,D) and pink with PAS (Figure 8A,B) was markedly increased in the Hilyses-treated groups compared with the control group. Figure 9 summarizes the results of villi length and crypt depth in both groups. In the treated group, the lengths of the intestinal villi and the lengths of the crypt depths showed significant increases compared to those of the control group.

### 3.6. Growth Performance and Feed Utilization in Nile Tilapia Fry and Fingerlings

Table 6 depicts the effect of Hilyses supplementation on growth performance parameters and feed utilization of tilapia fry. Overall, the main effects of both factors (Hilyses and broodstock on Hilyses) were significant (*p* < 0.05) for all growth parameters of *O. niloticus*. Meanwhile, the interactions between the main effects were not significant (*p* > 0.05) for the growth parameters. Hilyses dietary supplementation (either in the broodstock diet, the fry diet, or in both the broodstock and fry diet) resulted in higher fish performance parameters (including BWG, FI, and SGR) compared with those in the C-C group (negative control group). These changes resulted in lowered feed conversion ratios for all Hilyses supplemented groups. Moreover, the highest improvement was recorded in the H-H group (both broodstock and fry diets supplemented with 0.4% Hilyses) compared with other supplemented groups; however, the performance parameters of both H-C and C-H were nearly similar.

## 4. Discussion

The reproductive performance of fish is influenced by many factors, e.g., nutrition, hatchery management and environmental conditions. Dietary supplementation of Hilyses in Nile tilapia broodstock showed higher seed production, immune response, and seed survival than the control group. In accordance with these findings, Abo-State and Tahoun [38] observed that 0.02% dietary supplementation with *S. cerevisiae* significantly improved both seed production (seed/m^3^) and seed/female comparatively to 0.01%; moreover, both 0.01 and 0.02% supplementations gave significantly higher values than the un-supplemented diet. In addition, Abasali and Mohamad [39] found a significant increase in fry production and fertility of Helleri females fed probiotics (containing *Bifidobacterium thermophilum*, *Lactobacillus casei*, *Enterococcus faecium*, and *L. acidophilus*) compared with non-supplemented females. Moreover, Li and Gatlin [40] first studied the nucleotide nutrition of broodstock haddock and observed that the first feeding success of larvae from nucleotide-fortified broodstock was significantly higher than that of larvae from broodstock fed a basal diet without supplemented nucleotides. In the present study, improved seed production of Nile tilapia may be related to the nutrients and bioactive materials in Hilyses that directly enhance broodstock nutrition and immunity. Izquierdo, et al. [15] stated that modification of the nutritional quality of broodstock diets in continuous spawners with short vitellogenic periods even during the spawning season improves gonadal development and seed quality.

For seed survival, significant differences were found in seeds obtained from the Hilyses-treated broodstock groups (H-H and H-C), which showed the highest rate, followed by the C-H group, and the lowest survival rate was recorded in the C-C group. Transferring maternally trained innate immune elements from the Hilyses-treated broodstock into the yolk may increase the resistance of seeds against diseases and make them more resilient to adverse environmental conditions. The survival and size of larvae from haddock broodstock fed a nucleotide-supplemented diet were greater than that of larvae from broodstock fed the basal diet [15]. The mechanisms by which dietary nucleotides, β-glucans, and MOS promote growth and survivability include the improvement of intestinal morphology and the reduction of energy required for nucleotide synthesis, which allows more energy for the metabolic functions needed for the development and growth of fish larvae [41].

Gut health is considered the main factor that could affect the growth, reproductive performance, and immune status of aquatic animals [42,43]. Histological findings of the intestinal tract showed an improvement in intestinal microstructure in terms of increased fold height, enterocyte height and microvilli length, as well as the number of goblet cells in the Hilyses-treated group compared to the control group. This finding is consistent with the results of Dimitroglou et al. [44] and Xu et al. [45], who found that dietary MOS and nucleotides improve intestinal structure and health. Moreover, modulation of intestinal immunity is very important to maintain fish health. Mucins produced by goblet cells act as a barrier between the external environment and the internal environment of the body, select and transport essential nutrients through the epithelium, eliminate deleterious pathogens, and protect the mucosal layer from dehydration [46]. The current study revealed that dietary inclusion of Hilyses increased the expression of the MUC-2 gene in the intestine by 12.4-fold and in the liver 9.75-fold. Midhun et al. [32] reported that dietary probiotics could modulate MUC-2 gene expression in *O. niloticus*. Additionally, Nieves-Rodríguez et al. [47] showed that the expression of MUC-2 in *Atractosteus tropicus* tended to increase with the addition of a prebiotic, β-1,3/1,6-glucan derived from yeast at 1.5%, while there was a decrease in the fish fed 2% of the prebiotic, which indicated that high concentrations of this immune stimulant inhibited the expression of MUC-2. The elevated IGF-1 RNA levels in the liver and intestine of the Hilyses-treated group confirmed its potential growth-promoting effect. Midhun et al. [32] stated that hepatic IGF-1 mRNA expression could alter the growth rate in juvenile salmon.

Hematological and biochemical parameters usually reflect fish health status [48]. The recorded significant increase in hematological indices (including MCV and MCH) with a nonsignificant difference in MCHC in the Hilyses-supplemented group indicates that Hilyses has a positive impact on erythrogram, which directly reflects the health and productive status of Nile tilapia. In a similar sense, Abu-Elala et al. [49] observed a significant increase in RBC count, WBCs count, Hb concentration and PCV percent in Nile tilapia fed *S. cerevisiae* byproducts. The improved erythrogram could be related to the hepatostimulatory and hepatoprotective effects of Hilyses. The values of RBCs, hematocrit, and Hb were increased in the Hilyses-supplemented group. These results probably are associated with the regulation of the metabolic rate and wellbeing of Nile tilapia induced by *S. cerevisiae* feeding. Increasing of RBCs and Hb causes oxygen availability in the body tissues, owing to the absence of anemic features. The balanced levels of RBCs, hematocrit, and Hb were also attributed to the role of dietary *S. cerevisiae* in regulating the immune system. The antioxidant influence of the *S. cerevisiae* result in the balance of the protection of RBCs membranes and increases their life span by defending them against harmful impacts of oxygen-free radicals (ROS) and alleviating anemia and membrane disruption, as well as reducing cell hemolysis and degeneration [50,51]. In this regard Dawood et al. [46] reported that Nile tilapia fed dietary *S. cerevisiae* had enhanced RBCs, hematocrit, and Hb indices. Concurrent with the hematological indices, the results of related hepatic enzymes (ALT and AST), renal function (urea and creatinine), and blood protein profile (total protein, albumin, and globulin) of Nile tilapia serum following feeding, with or without Hilyses, refer to the stable metabolic rate under the current study conditions without apparent impairment of the fish health status [52].

The oxidative stress occurs during stressful environmental conditions, and pathogenic invasions that liberate ROS due to the imbalance of free radicals production and removal [53]. Excessive ROS also induces lipid peroxidation and damages RNA, which can be measured by the level of malondialdehyde (MDA) [54]. The total serum antioxidant capacity and GSH activity in the liver and intestine of fish fed Hilyses showed increased levels, while the MDA level was decreased which probably associated with the radical scavenge potential of the functional substances (nucleotides, β-glucans, and MOS) [55]. Similarly, Abu-Elala et al. [5] stated that the antioxidative response and MDA level were lowered in fish fed dietary *S. cerevisiae*.

High levels of cortisol indicate that the organism is suffering from stressful conditions and requires high glucose levels to afford the energy needed to cope with the stress [56,57]. Interestingly, the obtained results highlighted that tilapia-fed Hilyses had reduced cortisol level compared to fish fed the basal diet. Probiotics and prebiotics are known for the antistress influence, which is attributed to their role in improving the immunity and antioxidative capacity of aquatic organisms [58,59].

The enhancement of the innate immunity offers an interesting and attractive approach to increase the disease resistance of broodstock, newly hatched fish larvae and the first feeding fish, especially since the adaptive immune system is not fully developed [60]. Dietary inclusion of Hilyses in broodstock feeds upregulated some immune-related genes, such as *TLR-2*, *IL-1β* and *TNF-α*, in the liver and intestinal tissues. Zhang, et al. [60] suggested that to induce trained innate immunity by β-glucans, several different receptors must be engaged, such as Dectin-1 and TLRs. Stimulation of broodstock with β-glucans or any pathogen-associated molecular pattern (PAMP) at low doses increased the potential not only to resist pathogens, but also to transfer trained innate immunity to offspring. In addition to the direct innate immune training of broodstock, the molecules that are known to induce trained innate immunity may be maternally transferred and taken up by developing oocytes during vitellogenesis, potentially increasing the innate defense of developing embryos/larvae while the fish embryo or larvae are still in the eggs.

Gut-associated lymphoid tissue (GALT) is a diffusely arranged epithelium and includes immune cells along the teleost gut. This tissue expresses various pattern recognition receptors (PRRs), such as TLRs, which show significant expression when engaged with β-glucans. The ligation of *TLR-2* with β-glucans led to the upregulation of proinflammatory cytokine genes, such as *TNF-α* and *IL-1β*, without any inflammatory signs in tissues. This finding may be due to the expression of anti-inflammatory cytokines and increased antioxidant enzyme activity [32]. Several articles have indicated that upregulation of proinflammatory cytokines improves immune promptness and increases disease resistance in tilapia [9,18,61].

Dietary fortification of Hilyses improved the growth performance and feed utilization of Nile tilapia fry. This finding may be due to the acceleration of digestive system maturation and to the increased nutrient digestion induced by the nutrients and bioactive compounds in Hilyses. Essential amino acids, such as glutamic acid, play a substantial role in tissue proliferation and regeneration that consequently improve nutrient digestion and feed utilization [62]. Modulation of intestinal microbiota by MOS increases villus integrity and resistance to pathogenic bacteria, which increases digestion and absorption efficiency [17,47,63]. β-glucans stimulate the proliferation of beneficial gut flora and eliminate pathogenic gut flora, resulting in increased weight gain [64]. In a similar sense, the use of prebiotics containing β-glucans and MOS improved the growth performance of Nile tilapia, common carp (*Cyprinus carpio*), beluga (*Huso huso*), and sea cucumbers (*Apostichopus japonicus*) [63,64,65,66,67]. In addition, the use of S. cerevisiae live cells (20 g/kg) markedly improved the growth performance and nutrient utilization in Nile tilapia broodstock [38]. Notably, the present study suggested the dose of 0.4% (4 g/kg diet) of Hilyses, whereas Abo-State and Tahoun [38] recommended a 20 g/kg diet of *S. cerevisiae* for enhancing the performance of tilapia broodstock. The low dose of Hilyses indicates that fermented yeast cell wall components (e.g., nucleotides, β-glucans, and MOS) in its pure form could be more effective than the incorporation of the whole yeast live cells.

## 5. Conclusions

In summary, boosting the nutrition and immunity of Nile tilapia broodstock during the spawning season by using dietary fermented yeast products improved fry production and survival. Dietary inclusion of Hilyses modulated some growth- and immune-related genes and improved the intestinal microstructure morphology. Furthermore, this treatment increased the antioxidant enzyme activity and reduced cortisol levels. Hilyses had major effects on the growth performance and feed utilization in fry, especially those obtained from the Hilyses-treated broodstock and fed 0.4% Hilyses over three months.

## Figures and Tables

**Table 1:** Ingredients (%) and chemical composition of the experimental diets.

Ingredient %	Diet 1 (36% CP ^5^)	Diet 2 (40% CP)	Diet 3 (30% CP)
Fish meal (67%)	21.8	23.55	16
Soybean meal (48%)	37.3	44.84	30.5
Yellow corn	29.18	21.33	38.22
Wheat flour	6.51	5.75	8.5
Soybean oil	3	3	3.5
Mono calcium phosphate (23.7%)	0.8	0.28	1.3
Calcium carbonate	0.42	0.34	0.87
Common salt	0.5	0.5	0.5
Premix ^1^	0.3	0.3	0.3
Vitamin C	0.1	0.1	0.1
DL-Methionine	0.08	0	0.2
BHT ^2^	0.01	0.01	0.01
Total	100	100	100
Calculated Analysis (%)			
Crude protein	36.04	40	30.03
Dry matter	90.18	90.41	89.9
Ash	8.65	8.75	8.2
Ether extract	8.4	8.63	8.39
Crude fiber	3.58	3.95	3.26
NFE ^3^	43.33	38.66	50.1
Gross energy (kcal/100 g) ^4^	460.67	466.08	454.46
Calcium	1.22	1.19	1.23
Total phosphorus	1.06	1	1.01
Methionine	0.76	0.75	0.75
Lysine	2.25	2.54	1.79
Threonine	1.44	1.61	1.174
Linoleic acid	2.36	2.22	2.78
Chemical Analysis (%)			
Crude protein	36.11	40.08	30.07
Dry matter	90.07	90.37	89.93
Ash	8.67	8.73	8.18
Ether extract	8.43	8.56	8.36
Crude fiber	3.61	3.98	3.37
Calcium	1.20	1.21	1.25
Total phosphorus	1.04	1.01	1.03

^1^ Each kg vitamin and mineral mixture premix contained vitamin A, 4.8 million IU; D3, 0.8 million IU; E, 4 g; K, 0.8 g; B_1_, 0.4 g; riboflavin, 1.6 g; B_6_, 0.6 g, B_12_, 4 mg; pantothenic acid, 4 g; nicotinic acid, 8 g; folic acid, 0.4 g; biotin, 20 mg; Mn, 22 g; Zn, 22 g; Fe, 12 g; Cu, 4 g; I, 0.4 g, selenium, 0.4 g; and Co, 4.8 mg. ^2^ Antioxidant–butylated hydroxytoluene. ^3^ Nitrogen-free extract. ^4^ Gross energy. ^5^ Crude protein. Based on 5.65 Kcal/g protein, 9.45 Kcal/g fat, and 4.1 carbohydrate Kcal/g [19].

**Table 2:** Environmental water parameters measured daily in the hatchery during the trial.

Parameters	March	April	May	June	July	August
Temperature (°C)	28 ± 1	28 ± 1	30 ± 1	30 ± 1	31 ± 1	32 ± 1
Dissolved oxygen (mg/L)	5.9 ± 0.2	6 ± 0.3	6.3 ± 0.2	6.2 ± 0.1	6.5 ± 0.2	6.5 ± 0.1
pH	8.3 ± 0.2	8.6 ± 0.2	8.6 ± 0.2	8.7 ± 0.1	8.7 ± 0.3	8.7 ± 0.2
Total ammonia (mg/L)	0.1 ± 0.02	0.1 ± 0.01	0.2 ± 0.02	0.2 ± 0.02	0.2 ± 0.01	0.2 ± 0.01

**Table 3:** Primer sequences and target genes for SYBR Green RT-PCR.

Genes	Primer Sequences	Reference
*EF-1α* (housekeeping)	CCTTCAACGCTCAGGTCATC	Gröner et al. [31]
	TGTGGGCAGTGTGGCAATC	
*IGF1*	CATCGTGGACGAGTGCTG	Midhun et al. [32]
	ACAGGTGCACAGTACATCTCAAG	
*TLR-2*	CCCACAATGGATTCACCAG	
	AAAGATCAAGACTCAAGGCACTG	
*MUC-2*	CAACTGTTTTTGAGACAACTTCAGA	
	CTGAAGTGACCGTGGAAGG	
*TNF alpha*	CCAGAAGCACTAAAGGCGAAGA	Standen et al. [33]
	CCTTGGCTTTGCTGCTGATC	
*IL-1β*	GCTGGAGAGTGCTGTGGAAGAACATATAG	Castro et al. [34]
	CCTGGAGCATCATGGCGTG	

**Table 4:** Hematological parameters of Nile tilapia broodstock fed Hilyses and the control diets.

Parameters	Control	Hilyses	*t*-Value	*p*-Value
RBCs count (×10^6^ cell/mm^3^)	1.52 ± 0.02	1.68 ± 0.05	−2.836	0.02 *
Hematocrit value (%)	30.80 ± 1.39	38.80 ± 1.62	−3.738	0.006 **
Hb (g/dL)	5.79 ± 0.15	6.93 ± 0.17	−4.950	0.001 **
MCV (fl)	202.95 ± 7.83	230.71 ± 3.14	−3.288	0.01 **
MCH (pg)	38.22 ± 0.85	41.29 ± 0.48	−3.134	0.01 **
MCHC (%)	18.92 ± 0.66	17.92 ± 0.45	1.241	0.25
WBCs count (×10^3^ cell/mm^3^)	61.60 ± 1.43	63.80 ± 0.80	−1.339	0.2
Granulocyte %	38.00 ± 0.45	35.40 ± 0.68	3.200	0.01 **
Lymphocyte %	58.00 ± 0.71	60.80 ± 0.86	−2.514	0.04 *
Monocyte %	4.00 ± 0.45	3.80 ± 0.37	0.343	0.74

The results are expressed as the mean ± S.E. (*n* = 10) and were analyzed statistically by independent samples *t* tests, * statistically significant (*p* < 0.05), ** statistically highly significant (*p* < 0.01).

**Table 5:** Serum biochemical parameters of Nile tilapia broodstock-fed Hilyses and the control diets.

Parameters	Control	Hilyses	*t*-Value	*p*-Value
ALT (U/L)	5.44 ± 0.43	4.54 ± 0.28	1.776	0.11
AST (U/L)	42.24 ± 3.66	38.58 ± 3.45	0.730	0.49
Total protein (g/dL)	3.10 ± 0.09	2.97 ± 0.14	0.762	0.47
Albumin (g/dL)	1.89 ± 0.05	1.71 ± 0.07	2.020	0.08
Globulin (g/dL)	1.21 ± 0.08	1.26 ± 0.11	−0.387	0.71
Urea (mg/dL)	2.82 ± 0.29	2.91 ± 0.22	−0.259	0.80
Creatinine (mg/dL)	0.36 ± 0.01	0.36 ± 0.01	−0.065	0.95

The results are expressed as the mean ± S.E. (*n* = 10) and were analyzed statistically by independent samples *t*-tests.

**Table 6:** Growth performance and feed utilization in Nile tilapia fry after three months.

Treatments			Variables					
Groups	Hilyses% ^1^	Broodstock on Hilyses	Final BW at 3 m, g	Total BW gain, g	Total Feed Intake/fish, g	Final FCR ^3^, g/g	Final PER ^4^, g/g	Final SGR ^5^
C-C	0.0	−	15.4 ^c^	15.3 ^c^	20.5 ^d^	1.3 ^a^	2.5 ^c^	5.4 ^c^
C-H	0.4	−	19.8 ^b^	19.7 ^b^	23.8 ^c^	1.2 ^b^	2.8 ^b^	5.7 ^b^
H-C	0.0	+	20.8 ^b^	20.7 ^b^	25.2 ^b^	1.2 ^b^	2.7 ^b^	5.7 ^b^
H-H	0.4	+	26.2 ^a^	26.0 ^a^	28.9 ^a^	1.1 ^c^	3.0 ^a^	5.9 ^a^
-	-	SEM ^2^	0.372	0.372	0.115	0.012	0.012	0.012
Main Effects								
Hilyses		0.0%	18.1 ^b^	17.9 ^b^	22.9 ^b^	1.3 ^a^	2.6 ^b^	5.5 ^b^
-		0.4%	22.9 ^a^	22.9 ^a^	26.4 ^a^	1.1 ^b^	2.9 ^a^	5.8 ^a^
-		SEM	0.256	0.256	0.082	0.008	0.008	0.008
Broodstock on Hilyses		−	18.3 ^b^	18.2 ^b^	22.7 ^b^	1.2 ^a^	2.6 ^b^	5.5 ^b^
-		+	24.4 ^a^	24.3 ^a^	27.7 ^a^	1.1 ^b^	2.9 ^a^	5.9 ^a^
-		SEM	0.259	0.259	0.082	0.008	0.008	0.008
*p*-Value								
Hilyses			<0.001	<0.001	<0.001	<0.001	<0.001	<0.001
Broodstock			<0.001	<0.001	<0.001	<0.001	<0.001	<0.001
Hilyses × broodstock on Hilyses			0.235	0.235	0.137	0.253	0.563	0.213
F-Value								
Hilyses			179.203	179.203	906.941	111.447	557.261	524.053
Broodstock			261.810	261.810	1810.735	93.995	468.853	764.349
Hilyses × broodstock on Hilyses			1.437	1.437	2.728	1.514	0.365	1.827

The results were statistically analyzed by using two-way analysis of variance (ANOVA) with type of diets and broodstock followed by Tukey’s HSD test for mean multi-comparison test. Significance was determined at *p* < 0.05. ^a, b, c^ Means within a column with different superscripts are significantly different (*p* < 0.05). ^1^ Hilyses^®^, nucleotides, MOS, and β-glucan (ICC Company, Brazil). ^2^ SEM: standard error of the mean. ^3^ Feed conversion ratio = feed intake (g)/body weight gain. ^4^ Protein efficiency ratio = weight gain (g)/protein intake (g). ^5^ Specific growth rate = (Ln. Final body weight–Ln. Initial body weight) × 100/experimental period.

## Data Availability

The data presented in this study are available on request from the corresponding author.
